# Foot and ankle characteristics of children with an idiopathic toe walking gait

**DOI:** 10.1186/1757-1146-5-S1-O14

**Published:** 2012-04-10

**Authors:** Cylie Williams, Paul Tinley, Michael Curtin

**Affiliations:** 1 Charles Sturt University, Albury, NSW, Australia; 2 Southern Health, Cardinia Casey Community Health, Cranbourne VIC, 3977, Australia; 3 Peninsula Health – Community Health, VIC,, 3977, Australia

## Background

Idiopathic toe walking (ITW) in children has been associated with ankle equinus, and while equinus has been linked with foot deformity in adults, there has been limited investigation on the impact of equinus on the structural foot change in children.

This study sought to use the weight bearing lunge test [1] and Foot Posture Index-6 [2] to evaluate the weight–bearing foot and ankle measures of children with an ITW gait and compare these to their age matched peers.

## Materials and methods

Sixty children between the ages of four and eight years were grouped into an ITW (N=30) and a non-toe walking (NTW) (N=30) cohort using a validated ITW tool. The ankle range of movement and FPI-6 was calculated during appropriate weight–bearing test and stance.

## Results

There was a highly significant difference in the weight–bearing lunge test measures between the ITW cohort and the NTW cohort. The FPI-6 comparison was not significant. The lunge test was also not predictive of the FPI-6 in the ITW cohort.

## Conclusion

Children with an ITW gait demonstrated reduced flexibility at the ankle joint but had similar weight–bearing foot posture when compared with NTW children. This shows that for children between the ages of 4 to 8 years, an ITW gait style impacts on the available dorsiflexion of the ankle but not the weight–bearing foot posture.
